# Impact of diapause on the cuticular hydrocarbons and physiological status of *Hippodamia variegata*

**DOI:** 10.1093/jisesa/ieae005

**Published:** 2024-02-22

**Authors:** Mahsa Khabir, Hamzeh Izadi, Kamran Mahdian

**Affiliations:** Department of Plant Protection, Faculty of Agriculture, Vali-e-Asr University of Rafsanjan, Rafsanjan, Iran; Department of Plant Protection, Faculty of Agriculture, Vali-e-Asr University of Rafsanjan, Rafsanjan, Iran; Department of Plant Protection, Faculty of Agriculture, Vali-e-Asr University of Rafsanjan, Rafsanjan, Iran

**Keywords:** diapause, *Hippodamia variegata*, cuticular hydrocarbons, glycogen, lipids

## Abstract

The spotted amber ladybird, *Hippodamia variegata* (Goeze) (Coleoptera: Coccinellidae), is known to be a potent predator of aphids, psyllids, whiteflies, mealybugs, and some butterfly species. This ladybeetle overwinters in the diapausing adult stage. The current study aimed to evaluate the impact of diapause on the energy resources and cuticular hydrocarbons (CHCs) of the female ladybeetle, specifically comparing the changes in glycogen, lipid, and protein contents, and CHCs profile of diapausing and non-diapausing adults. In this study, gas chromatography-mass was used to analyze whole-body extracts of the beetles. Results showed no significant differences between the amount of glycogen, lipid, and protein contents of diapausing and non-diapausing ladybeetle. The CHCs profile of *H. variegata* consisted of 24 hydrocarbons categorized into 2 groups: linear aliphatic hydrocarbons (*n*-alkanes) and methyl-branched hydrocarbons (17 molecules), as well as unsaturated cyclic compounds (7 molecules). The *n*-alkanes, with 14 compounds, were identified as the primary constituents of the CHCs of the ladybeetle. Six molecules were common to non-diapausing and diapausing beetles, 5 were exclusive to non-diapausing beetles, and 13 were exclusive to diapausing beetles. Moreover, we noted a significant difference in the quantity and quality of CHCs between diapausing and non-diapausing beetles, with diapausing beetles synthesizing more CHCs with longer chains. This disparity in CHC profiles was concluded to be an adaptation of *H. variegata* to survive harsh environmental conditions during diapause.

## Introduction

Diapause in insects is a neurohormonal-mediated condition of low metabolic activity characterized by the cessation of development and reproduction. It is usually triggered by environmental cues such as photoperiod and temperature, and it occurs at a specific developmental stage that varies among species (Gill et al. 2017, Denlinger 2022). Females in numerous insect species undergo adult reproductive diapause to prepare for adverse conditions and delay sexual maturity and reproduction until the next growing season (Guo et al. 2021, Karp 2021, Kurogi et al. 2023). Diapause is characterized by increased energy storage, reduced metabolism, and enhanced resistance to water loss and low temperatures. The primary challenge for diapausing insects is their ability to survive extended periods without food. Therefore, managing metabolic resources becomes crucial when food sources are scarce, and insects employ 2 primary strategies to deal with this challenge: accumulating reserves before entering diapause and reducing metabolic activity during diapause (Denlinger 2009, Hahn and Denlinger 2010, Gill et al. 2017, Saunders 2020). Two primary functions of insect cuticular hydrocarbons (CHCs) are to shield insects from drying out and to act as chemical signals for communication within the same species. It is hypothesized that this dual purpose is closely linked to the components of CHCs. Several insect species have been found to have the ability to change their CHC compositions throughout their lifetime in response to various environmental conditions (Drijfhout et al. 2009, Liebig et al. 2009, Würf et al. 2020). CHCs play a multifaceted role in diapause, including chemical communication, physiological adaptation, and protection against environmental stressors. Understanding the specific CHC profiles and their functions in different insect species can provide valuable insights into the mechanisms and regulation of diapause (Ala-Honkola et al. 2020, Holze et al. 2021, Kárpáti et al. 2023).

CHCs typically consist of a complex mixture of *n*-alkanes, methyl-branched alkanes, and unsaturated hydrocarbons. CHCs form thin hydrophobic coatings on the cuticular surfaces of most insects and other arthropods (Buckner et al. 1996, Howard and Blomquist 2005, Drijfhout et al. 2009, Liebig et al. 2009, Blomquist 2010, Stinziano et al. 2015, Ala-Honkola et al. 2020). Cuticle surface lipids in a particular species often consist of hydrocarbons, ranging from a few to over 100 components, with carbon chains varying from 21 to 50 and accounting for more than 90% (Blomquist 2010, Menzel et al. 2017a, Blomquist et al. 2018). CHC mixtures can vary in structural characteristics, such as overall chain length (mostly between C20 and C45), quantity, and locations of methyl branches and double bonds, the chirality of methyl-branched carbons, and E/Z stereochemistry of double bonds and their structure ranges from simple to extremely complicated. Furthermore, these compounds serve multiple functions (Estrada-Peña et al. 1995, Jennings et al. 2014, Adams et al. 2021, Blomquist and Ginzel 2021). CHCs may play a varying role in chemical communication among populations of a species, potentially affecting the speciation process and influenced by interactions with closely related species (Jennings et al. 2014, Würf et al. 2020, Adams et al. 2021). Moreover, CHCs have multiple functions in the mutualistic relationship between insects and microbes. A recent study revealed the presence of 3 alkyl amides exclusively in the CHC profiles of leafcutter ants that coexist with symbiotic bacteria. These alkyl amides may play a role in the tripartite symbiosis of leafcutter ants (*Atta sexdens*, *A. cephalotes*, and *Acromyrmex octospinosus*) (Fladerer et al. 2023). The composition of CHC is heritable and primarily controlled by genetics. However, various environmental factors, including food availability, host species, and short-term acclimation to climatic conditions, can also influence it (Menzel et al. 2017b).

Insects usually employ a variety of adaptations, such as suppression of metabolism and reduction of activity, to cope with dehydration stress and maintain water homeostasis during overwintering. Moreover, changes in CHCs may also reduce water loss. For example, the diapausing adults of *Drosophila montana* had a higher concentration of long-chain hydrocarbons and a lower concentration of short-chain hydrocarbons on their cuticles compared to the non-diapausing flies (Ala-Honkola et al. 2020). In diapausing females of the northern house mosquito, *Culex pipiens*, the production of extra-CHCs is associated with reduced water loss (Benoit and Denlinger 2007a). Based on the chain length, the CHCs of different insects can be divided into 2 distinct groups. The first group consists of low molecular weight alkanes, monomethyl alkanes, and olefins (unsaturated hydrocarbons with double bonds) with chain lengths ranging from C23 to C27. The second group comprises higher molecular weight olefins or methyl-branched compounds with a chain length ranging from C37 to C45 (Batista-Pereira et al. 2004).

The spotted amber lady beetle, *Hippodamia variegata* (Goeze) (Coleoptera: Coccinellidae), is also known as the Variegated ladybug or Adonis ladybird. It can be found in fields, pastures, various plant biotopes, bushes, and coastal areas. Adults and larvae feed on aphids, psyllids, whiteflies, mealybugs, and some species of butterflies, including flour moths. Like other insect species, the growth of this insect is strongly influenced by environmental conditions, such as food supply and temperature. *H. variegata* is a multivoltine species that survives the winter as diapausing adults under the bark of host plants (Kontodimas and Stathas 2005, Jafari 2011).

Overwintering insects experience prolonged exposure to low temperatures, starvation, and desiccation, which triggers complex adaptive changes in their physiological and biochemical regulation (Denlinger 2002, Xiao et al. 2022). Therefore, in the current study, we investigated the correlation between the levels of water, lipid, glycogen, and protein in diapausing and non-diapausing adults of *H. variegata*. Additionally, we used gas chromatography-mass spectrometry to analyze whole-body extracts of diapausing and non-diapausing beetles, aiming to identify and measure the CHC profile. The goal was to comprehend the impact of diapause on the ladybird beetles’ CHCs. We predicted that diapause triggers alterations in the biochemical contents of beetles and that the CHCs of diapausing beetles consist of longer-chain hydrocarbons compared to non-diapausing ones. This adaptation should increase their chances of survival during the overwintering period. However, our findings revealed that the second prediction held while the first one did not follow the expected trend.

## Material and Methods

### Collection of Ladybug Beetles

*Hippodamia variegata* adults were captured from pistachio orchards in Kerman, Kerman province, Iran (30° 16’ 59.56‘ N, 57° 04’ 43.64’ E). They were subsequently housed in 15 × 15 × 15 cm plastic breeding containers with a 5 cm opening diameter on the lid, which was covered with lace for proper ventilation. To study the variations in biochemical contents throughout the year, adult insects were collected monthly during the first 6 months (usually in the middle of each month and 24–48 h before the experiment) from the gardens. To investigate the changes in CHCs and water content of diapausing and non-diapausing adult insects, samples (male and female adults) were collected on the 15th of January and July (2021–2022) from pistachio orchards. In January, when the air temperature drops, ladybirds become scarce. To overcome this problem, many adult ladybirds were collected in September from their hibernation shelters. They were then placed in plastic breeding containers and kept in the garden where they were originally found, under natural environmental conditions.

### Body Water Content Assay

In mid-July (the hottest month) and January (the coldest month), 5 adult insects were gathered and weighed to determine their fresh mass (FM) and water mass (WM). Once the FM was measured, the insects were individually placed in 5-ml plastic tubes with pre-perforated walls. To determine the dry mass (DM), the tubes containing the insects were placed in an oven set at 60 °C for 24 h. Subsequently, the insects were weighed using an analytical scale (with an accuracy of 0.0001). The amount of weight lost during the drying process was used to calculate the WM. This method provides an estimate of the water content present in the insect’s body at the time of sampling. It is important to ensure that the drying process is conducted under conditions that do not result in significant loss of any other volatile components, such as lipids or amino acids, which could affect the accuracy of the measurement. However, weight loss is an indicator of water mass and was calculated as follows (Feng et al. 2016): %WM = ((FM − DM))/(FM) × 100.

## Biochemical Changes Analysis

### Glycogen Content Assay

To estimate the glycogen content of the insects, the following steps were taken: First, in the middle of each month from the bulk containers, 5 insects were weighed individually and then homogenized in 200 µl of 2% sodium sulfate (Na_2_SO_4_) and 1,300 µl of chloroform: methanol (1:2). After centrifugation at 7,150 rpm for 10 min, the resulting pellet was washed with 400 µl of 80% methanol. Subsequently, 250 µl of distilled water was added to the pellet and the solution was heated at 70 °C for 5 min. Next, 1 ml of anthrone reagent (500 mg anthrone dissolved in 500 ml concentrated H_2_SO_4_) was added to 200 µl of the resulting solution, which was then heated at 90 °C for 10 min. Finally, the optical density of the solution was measured at 630 nm using a spectrophotometer (T60U, Harlow Scientific, USA), and the glycogen concentration was estimated using a standard glycogen curve. This method allows for the determination of glycogen content in insect samples using a series of chemical reactions and measurements (Heydari and Izadi 2014). Each month, the experiments were conducted 5 times with individual insects.

### Total Lipid Content Assay

To determine the total lipid content, 300 µl of the supernatant obtained from the previous centrifugation step was evaporated in an oven at 35 °C. Next, 300 µl of H_2_SO_4_ was added to each tube, and the tubes were heated at 90 °C for 10 min before being allowed to cool. After cooling, the mixture was stirred and 2,700 µl of vanillin reagent (600 mg vanillin, 100 ml distilled water, and 400 ml of 85% H_3_PO_4_) was added to each tube. The tubes were then kept at room temperature for 30 min. The amount of lipids present in each sample was measured spectrophotometrically at 530 nm using a spectrophotometer (T60U, Harlow Scientific, USA). To calculate the amount of lipids, present in each sample, an absorption standard curve was constructed using triolein. This method involves a series of chemical reactions and measurements to determine the total lipid content of the insect samples (Warburg and Yuval 1997). The experiment was conducted 5 times, with individual insects each month.

### Total Protein Content Assay

To determine the total protein content of the insects, 5 adults were collected from the bulk container in the middle of each month. The adults were weighed individually and then homogenized using 1.5–2 ml of 80% ethanol. After homogenization, the mixture was centrifuged for 10 min at 12,000 rpm. To measure the protein content, 0.05 ml of the supernatant obtained from the centrifugation was mixed with 1.5 ml of a reagent consisting of 100 mg of Coomassie Brilliant Blue, 50 ml of ethanol, and 100 ml of 85% H_3_PO. The absorption of the resulting solution was measured at 595 nm using a spectrophotometer (T60U, Harlow Scientific, USA). In this method, bovine serum albumin protein was used as a standard for comparison. This process involves a series of chemical reactions and spectrophotometric measurements to determine the total protein content of the insect samples (Bradford 1976). The experiment was conducted 5 times, with individual insects each month.

### Extraction of Cuticular Hydrocarbons (CHCs) and GC–MS Analysis of CHCs

Insects (2 sets of 3 adults each month) were placed in a glass test tube. Then, 300 µl of a non-polar solvent, *n*-hexane, was added to each tube. Then, the lid of each tube was closed with cellophane to prevent evaporation of the solvent. The tubes were shaken for 2 h. Finally, the contents of each tube were filtered using Whatman filter paper No. 1 and transferred to other glass test tubes with screw caps. Following that, the lids of the tubes were properly covered with parafilm to prevent any potential solvent evaporation. A test tube containing *n*-hexane solvent was used as a blank. The samples were stored in a refrigerator at a temperature of 4 °C until they were transferred to the central laboratory of Tehran University. The CHC profile of adult ladybugs was identified using gas chromatography–mass spectrometry. The specifications of the gas chromatography device used are as follows: the manufacturer of the device was Agilent Technologies, and the carrier gas used was helium, which flowed at a rate of 1 ml/min. The samples were injected into the injector in a volume of 0.6 µl at a temperature of 250 °C. The final time was 10 min, and the final temperature was 270 °C. The type of column was Rtx 5 MS, and the data were processed by Agilent MSD Chemstation (Rev E.02.02.1413). After obtaining the results, we eliminated the hydrocarbons that were present in both the control (*n*-hexane) and the samples. Then, we compared the samples to determine the presence or absence of hydrocarbons in the cuticle of both diapausing and non-diapausing ladybugs. The cuticular compounds were identified, quantified, and compared using retention indices and mass spectra. The experiment was conducted twice a month, with 3 individual insects each time.

### Statistical Analyses

The data obtained from the experiments (protein and glycogen content assays) were analyzed using a one-way analysis of variance (ANOVA) in SPSS 26.0. The statistical difference between the means of protein and glycogen contents was assessed using the LSD test at a significance level of 5%. The Student *t*-test was also used to compare the means of 2 different groups (body water content assay). This approach involves statistical tests to determine if there are significant differences between the means of the different groups studied.

## Results

**Fig. 1.**
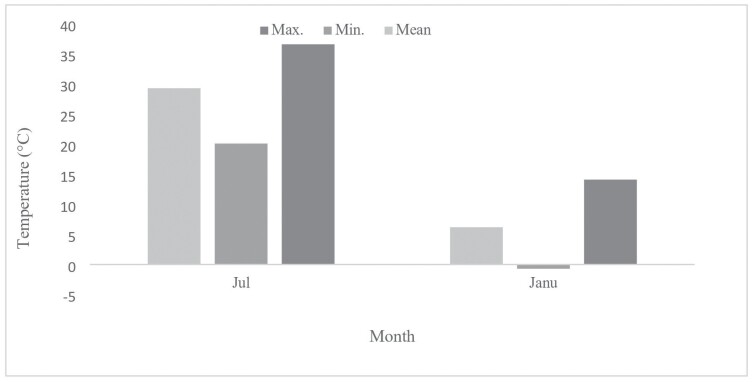
Temperature variations in Kerman from July 2021 to January 2022, including the average, lowest, and highest ambient temperatures (Meteorological Organization of Kerman, Iran). 106 × 65 mm (300 × 300 DPI).

**Fig. 2.**
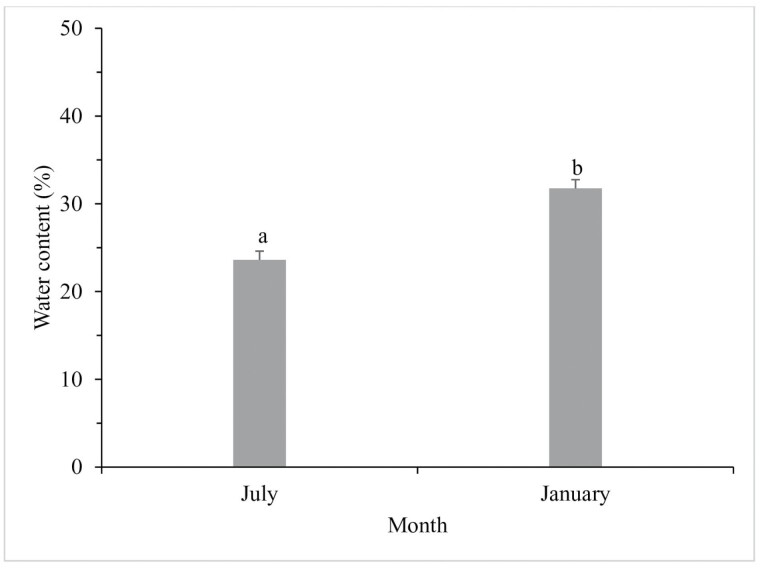
Change in the water content of *Hippodamia variegata* during January (diapausing)/July (nondiapausing). The presence of different letters above each column indicates a significant difference in the average water content. The error bar represents the standard error (*P* = 0.05) (Student *t*-test). Each month, the experiments were conducted 5 times, with individual insects. 116 × 97 mm (300 × 300 DPI).

Figure 1 presents the seasonal fluctuations in the average, minimum, and maximum ambient temperatures in Kerman during January 2021 and July 2022. The coldest temperature was recorded in January, while the hottest temperature occurred in July. As depicted in Fig. 2, the water content of diapausing beetles (31.7%) was significantly higher than that of non-diapausing ones (23.6%) (*F*_1, 8_ = −4.34, *P* = 0.002).

**Fig. 3.**
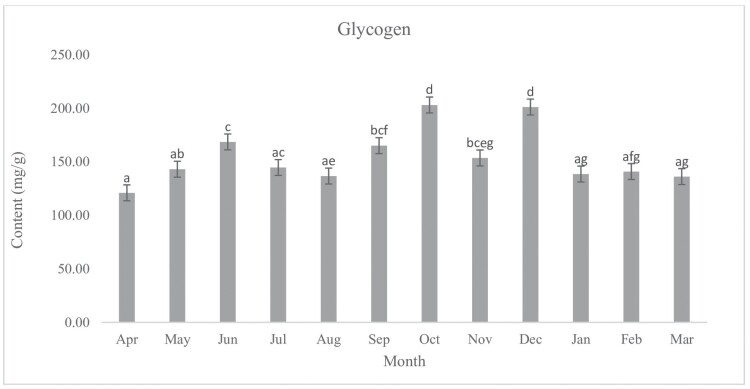
Change in the glycogen content of *Hippodamia variegata* during different months of 2021–2022. The presence of distinct letters above each column indicates a significant variation in the average glycogen content. The error bar represents the standard error (*P* = 0.05) (LSD test). Each month, the experiments were conducted 5 times with individual insects. 114 × 85 mm (300 × 300 DPI).

**Fig. 4.**
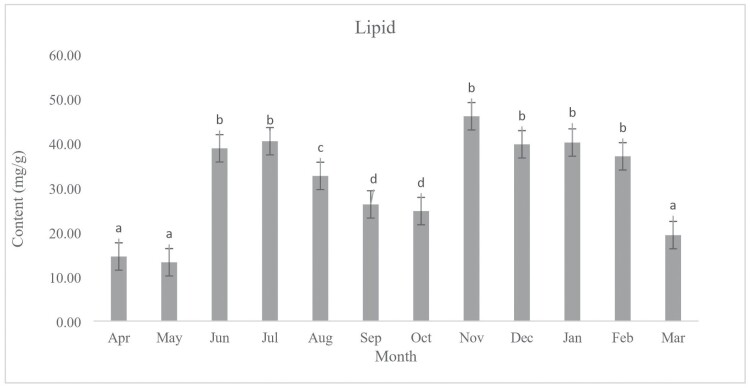
Change in the lipid content of *Hippodamia variegata* during different months of 2021–2022. The presence of distinct letters above each column indicates a significant variation in the average lipid content. The error bar represents the standard error (*P* = 0.05) (LSD test). The experiment was conducted 5 times, with individual insects each month. 114 × 84 mm (300 × 300 DPI).

**Fig. 5.**
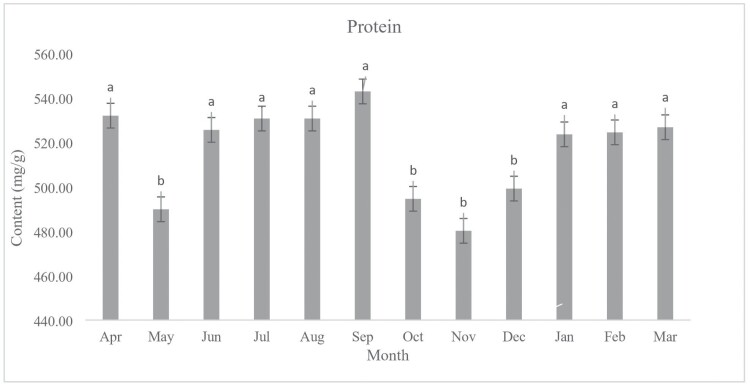
Change in the protein content of *Hippodamia variegata* during different months of 2021–2022. The presence of distinct letters above each column indicates a significant variation in the average protein content. The error bar represents the standard error (*P* = 0.05) (LSD test). The experiment was conducted 5 times, with individual insects each month. 115 × 85 mm (300 × 300 DPI).

During the period of diapause from April 2021 to May 2022, the chemical contents were measured monthly. Our results indicate no significant differences between the amount of glycogen (*F*_11, 48_ = 8.89, *P* < 0.001) content of diapausing (January) and non-diapausing (July) ladybeetle (Fig. 3). The amount of glycogen present in October and December was significantly higher compared to the other months. There were no significant differences in the lipid content of diapausing (January) and non-diapausing (July) ladybeetles (*F*_11, 48_ = 63.96, *P* < 0.001) (Fig. 4). In January and July, the protein content of diapausing and non-diapausing ladybeetles was not significantly different (*F*_11,48_ = 0.76, *P* = 0.68) (Fig. 5).

Based on chain length and chemical composition, our results showed a wide range of distinct compounds in the cuticle of the ladybeetles. These compounds include linear straight-chain (aliphatic) hydrocarbons, mono- and polycyclic (aromatic) hydrocarbons, and acyclic-branched hydrocarbons. However, we classified the CHCs of *H. variegata* into 2 main groups. (i) Linear aliphatic hydrocarbons (saturated and so-called *n*-alkanes) and a specific group of *n*-alkanes, methyl-branched hydrocarbons, with one or more methyl groups (CH3). Examples of these hydrocarbons include phytane, squalane, undecane, pentadecane, octadecane, dotriacontane, pentacosane, octacosane, heptacosane, octane, icosane, heptadecane, phytane, squalane, undecane, and methylheneicosane. These CHCs were identified as the main constituents of the cuticular extract of *H. variegata*; (ii) unsaturated compounds (olefins) with 1 (alkenes or monoenes), 2 (alkadiene or dienes), or 3 (alkatrienes or trienes) double bonds, i.e., xylene, phenol, limonene, diethyl phthalate, ribitol pentaacetate, gibberellic acid, and methyl dihydro jasmonate (Table 1). In addition to straight-chain hydrocarbons, our results indicate the presence of several mono-, di-, and polycyclic compounds. However, we did not find any compounds with more than 32 carbon atoms in our analysis. Dotriacontane, with 32 carbon atoms, was the heaviest constituent of the CHCs of *H. variegata*, while octane, xylene, and limonene, with C ≤ 10, were the lightest. The results obtained by comparing the CHC profiles of the non-diapausing (July) and diapausing (January) ladybirds are illustrated in Table 1. Results indicated that only 6 compounds, namely, octane (a low-molecular-weight and volatile hydrocarbon), xylene (an aromatic low-molecular-weight hydrocarbon), phenol (an aromatic organic compound), limonene (an aliphatic hydrocarbon), diethyl phthalate (a phthalate ester), and eicosane (an alkane, acyclic branched or unbranched hydrocarbons) are found in both diapausing and non-diapausing insects. Our results revealed that diapausing ladybird beetles of *H. variegata* produce longer-chain CHCs than non-diapausing ones. The compounds with more than 25 carbon atoms, such as squalene (a polyunsaturated hydrocarbon), dotriacontane, pentacosane, octacosane, and heptacosane, were found exclusively in diapausing beetles.

**Table 1.** The common cuticular hydrocarbons of *Hippodamia variegata*

No	Retention time (min)	Name	Formula	MW (g/mol)	IUPAC name	Presence
1	4.12	Octane (Acyclic—Alkanes)	C_8_H_18_ or CH_3_(CH_2_)_6_CH_3_	114.23	Octane	January (diapausing)/July (non-diapausing)
2	5.9	*p*-Xylene *m*-Xylene Xylene (Cyclic—Hydrocarbons, Aromatic—Benzene derivatives)	C_8_H_10_ or C_6_H_4_(CH_3_)_2_	106.16	1,4-Xylene 1,3-Xylene	January (diapausing)/July (non-diapausing)
3	9.05	Phenol, 2,2’-[(1-methyl-1,2-ethanediyl)bis(nitrilomethylidyne)]bis-	C_17_H_18_N_2_O_2_	282.34	N,N’-Bis(Salicylidene)-1,2-Propanediamine or Disalicylalpropylenediimine	January (diapausing)/July (non-diapausing)
4	11.79	dl-Limonene l-Limonene (Cyclic—Hydrocarbons, Alicyclic—Cycloparaffins—Cyclohexenes—Cyclohexenes)	C_10_H_16_	136.23	1-Methyl-4-prop-1-en-2-ylcyclohexene	January (diapausing)/July (non-diapausing)
5	36.09	Diethyl phthalate (Carboxylic acids—Phthalic acids)	C_12_H_14_O_4_ or C_6_H_4_(COOC_2_H_5_)_2_	222.24	Diethyl benzene-1,2-dicarboxylate	January (diapausing)/July (non-diapausing)
6	39.23	Eicosane (Hydrocarbons, Acyclic—Alkanes)	C_20_H_42_	282.5	Icosane	January (diapausing)/July (non-diapausing)
7	39.23	Heptadecane (Acyclic)	C_17_H_36_	240.5	Heptadecane	July (non-diapausing)
8	36.27	Ribitol pentaacetate	C_15_H_22_O_10_	362.33	[(2S,4R)-2,3,4,5-Tetraacetyloxypentyl] acetate	July (non-diapausing)
9	41.45	3,4-Dihydro-6,7-dimethoxyisoquinoline 2-oxide N-Cyano-N’,N’,N’’,N’’-	C_11_H_13_NO_3_	207.23	6,7-Dimethoxy-2-oxido-3,4-dihydroisoquinolin-2-ium	July (non-diapausing)
10	41.63	Gibberellic acid (Terpenes—Diterpenes—Diterpene Alkaloids—Diterpenes, Kaurane—Gibberellins)	C_19_H_21_O_6_	346.4	(1R,2R,5S,8S,9S,10R,11S,12S)-5,12-dihydroxy-11-methyl-6-methylidene-16-oxo-15-oxapentacyclo [9.3.2.15,8.01,10.02,8] heptadec-13-ene-9-carboxylic acid	July (non-diapausing)
11	41.63	3-Methylheneicosane	C_22_H_46_	310.6	3-Methylhenicosane	July (non-diapausing)
12	38.01	Methyl dihydrojasmonate (Cyclic—Hydrocarbons, Alicyclic—Cycloparaffins—Cyclopentanes)	C_13_H_22_O_3_	226.31	Methyl 2-hexyl-3-oxocyclopentane-1-carboxylate	January (diapausing)
13	39.22	Hexadecane (Acyclic—Alkanes)	C_16_H_34_	226.44	*n*-Hexadecane	January (diapausing)
14	39.36	Phytane (Terpenes—Diterpenes)	C_20_H_42_	282.5	2,6,10,14-Tetramethylhexadecane	January (diapausing)
15	39.39	Squalane (Acyclic—Alkenes—Polyenes)	C_30_H_62_	422.8	2,6,10,15,19,23-Hexamethyltetracosane	January (diapausing)
16	39.39	Decane, 2-methyl-(Acyclic—Alkanes)	C_11_H_24_	156.31	Undecane	January (diapausing)
17	39.39	Pentadecane, 2,6,10,14-tetramethyl- or Pristane (Terpenes)	C_19_H_40_	268.5	2,6,10,14-Tetramethylpentadecane	January (diapausing)
18	41.44	Octadecane (Acyclic—Alkanes)	C_18_H_38_	254.5	Octadecane	January (diapausing)
19	41.46	*n*-Dotriacontane (Acyclic—Alkanes)	C_32_H_66_	450.9	Dotriacontane	January (diapausing)
20	41.46	*n-*Pentacosane (Acyclic—Alkanes)	C_25_H_52_	352.7	Pentacosane	January (diapausing)
21	41.46	Octacosane (Acyclic—Alkanes)	C_28_H_58_	394.8	Octacosane	January (diapausing)
22	41.46	*n*-Heptacosane (Acyclic—Alkanes)	C_27_H_56_	380.7	Heptacosane	January (diapausing)
23	44.72	Cyclodecasiloxane, eicosamethyl-	C_20_H_60_O_10_Si_10_	741.5	2,2,4,4,6,6,8,8,10,10,12,12,14,14,16,16,18,18,20,20-icosamethyl-1,3,5,7,9,11,13,15,17,19-decaoxa-2,4,6,8,10,12,14,16,18,20-decasilacycloicosane	January (diapausing)
24	46.89	6-Aza-5,7,12,14-tetrathiapentacene	C_17_H_9_NS_4_	355.5	4,11,15,22-Tetrathia-2-azapentacyclo[12.8.0.03,12.05,10.016,21]docosa-1,3(12),5,7,9,13,16,18,20-nonaen	January (diapausing)

## Discussion

The observed increase in water content during diapause may be related to the maintenance of cellular hydration and protection against desiccation in these ladybeetles (Hodek 2003, Denlinger 2009, Denlinger 2022). In general, and contrary to our results, diapausing insects tend to have a lower water content than non-diapausing insects (Gill et al. 2017). This is because water is a critical component of metabolic processes, and reducing metabolic activity during diapause can lead to a decreased need for water. Additionally, some diapausing insects may reduce water loss by entering a state of reduced water exchange with the environment, which helps to conserve water resources (Benoit and Denlinger 2007b). However, contrary to our findings, it is uncommon to observe an increase in water content during diapause. Several studies have documented a decline in water content during the period of arrested development (Han and Bauce 1993, Wipking et al. 1995, Heydari and Izadi 2014). Conversely, certain insects have shown relatively stable water content during diapause (Adedokun and Denlinger 1985). However, based on our knowledge, this finding is an exception to the general trend, and further research is needed to fully understand the mechanisms behind diapause and its effects on water regulation in different species.

Our findings suggest that certain biochemical traits remained consistent during diapause and were not significantly affected by changes in temperature. This suggests that the organism has adapted to endure and overcome the challenges of diapause, especially with changes in the studied biochemical compounds. During diapause, many physiological processes slow down, including protein synthesis and degradation. As a result, the protein content of an insect may change during this period. In some cases, an increase in protein content has been observed during diapause (Adedokun and Denlinger 1985, Heydari and Izadi 2014, Khanmohamadi et al. 2016). The rise in protein content could be a reaction to stress caused by low temperatures (King and MacRae 2014). Alternatively, the proteins may be stored to be utilized when the insect emerges from diapause. On the other hand, a decrease in protein content has also been observed during diapause in some insects (Xiao et al. 2022). This may be because the organism is breaking down proteins for energy production during this period of suspended development. Overall, the relationship between protein content and diapause is complex and can vary depending on the organism and the specific conditions of diapause. Many insects accumulate lipid reserves in preparation for diapause, which serves as a source of energy during the dormant period (Xiao et al. 2022). The presence of glycogen in insects is crucial for regulating diapause, serving as both an energy source and a protective agent against freezing. Additionally, the amount of glycogen can influence the end of diapause. When the environment becomes favorable, insects can utilize stored glycogen to support the metabolic activities required for resuming growth and reproduction (Wipking et al. 1995, Hahn and Denlinger 2007, Zhou and Miesfeld 2009, King et al. 2020).

Based on our results, *n*-alkanes and methyl-branched hydrocarbons were found to be the major constituents of CHCs of *H. variegata*. In this regard, a combination of *n*-alkanes, *n*-alkenes, 3-methylalkanes, and monomethyl- and dimethyl-branched alkanes was discovered in the cuticle of *Tenebrio molitor* and *Tenebrio obscurus*. The critical point is that the chain length, the presence, number, and position of double bonds, as well as the number and position of methyl groups, broadly impact the chemical properties and physiological functions of the CHCs. However, several studies have revealed the significant impact of species, sex, developmental stage, body parts, and diapause on the CHC profiles of insects (Ala-Honkola et al. 2020, Sprenger et al. 2021, Kula et al. 2023). For example, 4 polyunsaturated alkenes were exclusively identified in the CHCs of the reproductive cast of the termite, *Zootermopsis nevadensis* (Isoptera: Archotermopsidae) (Liebig et al. 2009). Studies on the impacts of ambient conditions and sexual dimorphism on CHCs of the jewel wasp, *Nasonia vitripennis* (Hymenoptera: Pteromalidae) revealed the presence of female-specific C31 mono- and C33 di-methyl-branched alkanes, which act as sex pheromones (Buellesbach et al. 2022). Analysis of the CHCs revealed the significant impact of sex, developmental stage, and mating status on the CHC profile of *Dasineura oleae* (Diptera: Cecidomyiidae) (Caselli et al. 2023). Interestingly, compounds found in both diapausing and non-diapausing adults of *H. variegata* are primarily low molecular weight semiochemical hydrocarbons that are presumed to be interspecific communication agents. The semiochemical role of CHCs has been extensively studied in various insects, especially social ones such as *Heterotermes tenuis* (Isoptera: Rhinotermitidae) (Batista-Pereira et al. 2004), Dawson’s burrowing bee, *Amegilla dawsoni* (Hymenoptera: Apidae, Anthophorini) (Simmons et al. 2003), and the parasitoid wasp, *Urolepis rufipes* (Würf et al. 2020). In the comparison between diapausing and non-diapausing beetles, 5 specific compounds were found in non-diapausing beetles, which increased to 13 in diapausing beetles. Diapausing ladybirds have synthesized and stored more hydrocarbons than non-diapausing ones. Likewise, the diapausing pupae of the Bertha armyworm, *Mamestra configurata* Walker, had 3 times as many CHCs as non-diapausing ones (Hegdekar 1979). Moreover, diapause increased the number of CHCs in the flesh fly, *Sarcophaga crassipalpis* (Yoder et al. 1992). Our results demonstrated that longer-chain CHCs are exclusively synthesized in diapausing ladybird beetles. These relatively high-molecular-weight hydrocarbons are most likely associated with the winter survival of the beetles. In *D. suzukii*, winter morphs produce longer-chain methyl-branched and unsaturated hydrocarbons compared to same-age summer morphs (Kárpáti et al. 2023). Analysis of CHCs in diapausing and non-diapausing flies of *D. montana* revealed that the diapausing flies exhibited higher levels of long-chain hydrocarbons compared to the non-diapausing flies (Ala-Honkola et al. 2020).

The 5 specific compounds found in non-diapausing adult females of *H. variegata* are heptadecane (an alkane, which is an acyclic-saturated hydrocarbon), ribitol pentaacetate (a pentose alcohol), 6,7-dimethoxy isoquinoline, gibberellic acid, and 3-methylheneicosane. Interestingly, these 5 compounds are naturally found in various plants. This indicates that these compounds are primarily present during the feeding stage and are indirectly derived from the host plants of the beetle’s prey. These compounds are likely used as semiochemical agents in inter- and intraspecific communications. For example, (Z)-8-heptadecene and pentadecane were reported as shared semiochemicals in European *Ophrys* orchids and their pollinators, *Argogorytes fargeii* (Hymenoptera: Crabronidae) (Bohman et al. 2020). In non-diapausing pupae of the large and small white butterflies, *Pieris brassicae* and *P. rapae crucivora*, more than 90% of the hydrocarbons were saturated (Kaneko and Katagiri 2004). Moreover, xylene, phenol, limonene, ribitol pentaacetate, diethyl phthalate, gibberellic acid, methyl dihydro jasmonate, cyclodecasiloxane, and tetracosamethyl-cyclododecasiloxane were identified as cyclic hydrocarbons in the cuticle. Analyses of the cuticle of a pteromalid wasp, *U. rufipes*, revealed that *n*-alkanes and methyl-branched alkanes are the main constituents of male and female CHCs (Würf et al. 2020). The hydrocarbons identified in the cuticle of *H. tenuis* were mainly restricted to linear alkanes ranging from C24 to C27 (Batista-Pereira et al. 2004). Alkenes and 2-methyl branched alkanes with a chain length of C23–C31 constitute the main components of the CHC of *Drosophila melanogaster* (Ala-Honkola et al. 2020). Several unsaturated hydrocarbons and methyl-branched alkenes have also been reported in certain hymenopteran species’ CHC profiles (Kather and Martin 2012, 2015).

In general, the findings indicate that the biochemical characteristics of *H. variegata* remained consistent during diapause and did not exhibit any notable alterations due to temperature variations. Moreover, in this study, 24 hydrocarbons were identified in the cuticle of *H. variegata* through GC-mass analysis. By comparing diapausing and non-diapausing beetles, it was revealed that diapause causes the beetles to synthesize more CHCs than non-diapausing ones. Moreover, the CHCs of diapausing beetles were more complex (long-chain methyl-branched components) than non-diapausing beetles. We speculate that the differences in CHC profiles between diapausing and non-diapausing adults of *H. variegata* are an adaptation to cope with the harsh environmental temperatures during diapause.
